# Supplementary figures and images for: The effects of dietary iron supplementation on bacterial infections in Manduca sexta larval hemolymph
Source: bioRxiv. 2026 Mar 25:2026.03.21.713330. Preprint. [Version 2] doi: 10.64898/2026.03.21.713330 (PMC13041954; doi:10.64898/2026.03.21.713330)

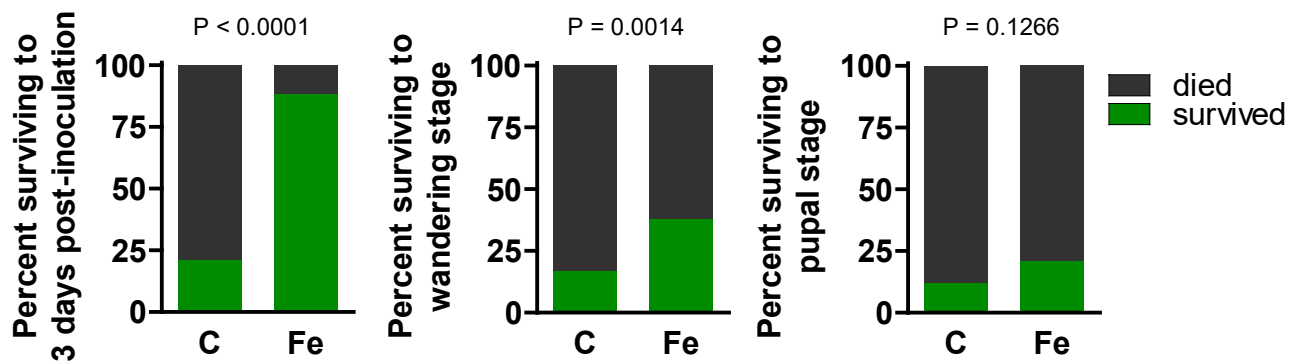

Supplement: Supplement 1 — S1 Fig. In the absence of antibiotics, iron supplementation decreased E. faecalis-induced mortality of feeding stage larvae. Larvae cultured on control (C) or iron-supplemented (Fe) diet without antibiotics were inoculated with E. faecalis at an OD600 of 0.02, and mortality was monitored. The percentage of larvae surviving at three days post-inoculation, the wandering stage, and the pupal stage are shown. A Fisher’s exact test was used to test for statistical significance (n = 24). [file media-1.pdf]
